# MyHEART: A Non Randomized Feasibility Study of a Young Adult Hypertension Intervention

**DOI:** 10.23937/2474-3690/1510016

**Published:** 2016-09-12

**Authors:** Heather M Johnson, Jamie N LaMantia, Ryan C Warner, Nancy Pandhi, Christie M Bartels, Maureen A Smith, Diane R Lauver

**Affiliations:** 1 Department of Medicine, University of Wisconsin School of Medicine and Public Health, USA; 2 Health Innovation Program, University of Wisconsin School of Medicine and Public Health, USA; 3 Department of Counselor Education and Counseling Psychology, Marquette University, USA; 4 Department of Family Medicine and Community Health, University of Wisconsin School of Medicine and Public Health, USA; 5 Department of Surgery, University of Wisconsin School of Medicine and Public Health, USA; 6 Department of Family Medicine, University of Wisconsin School of Medicine and Public Health, USA; 7 Department of Population Health Sciences, University of Wisconsin School of Medicine and Public Health, USA; 8 University of Wisconsin-Madison School of Nursing, USA

**Keywords:** Hypertension, Young adult, Feasibility, Self-management, Health coach, Primary care, Self-determination theory

## Abstract

**Background:**

In the United States, young adults (18–39 year-olds) have the lowest hypertension control rates (35%) compared to middle-aged (58%) and older (54%) adults. Ambulatory care for hypertension management often focuses on medication with little time for self-management and behavioral counseling. This study was designed to evaluate the feasibility of MyHEART, a telephone-based health coach self-management intervention for young adults. The goals were to determine the intervention’s ability to: 1) recruit young adults with uncontrolled hypertension, 2) maintain ongoing communication between the coach and participants, 3) increase participants’ engagement in self-management, 4) document coach-patient communication in the electronic health record, and 5) assess patient acceptability.

**Methods:**

Eligible participants were identified through the electronic health record. Inclusion criteria included 18–39 year-olds, with ICD-9 hypertension diagnoses and uncontrolled hypertension (≥ 140/90 mmHg), receiving regular primary care at a large multispecialty group practice. The intervention consisted of 6 telephone self-management sessions by a health coach targeting lifestyle modifications. Patients completed an open-ended acceptability survey.

**Results:**

Study uptake was 47% (9 enrolled/19 eligible). Mean (SD) age was 35.8 (2.6) years, 78% male, and 33% Black. Over 85% of enrolled young adults maintained communication with their health coach. At baseline, 11% reported checking their blood pressure outside of clinic; 44% reported blood pressure monitoring after the study. All coach-patient encounters were successfully documented in the electronic health record for primary care provider review. Open-ended responses from all surveys indicated that participants had a positive experience with the MyHEART intervention.

**Conclusions:**

This study demonstrated that MyHEART was feasible and acceptable to young adults with uncontrolled hypertension. Health coaches can effectively maintain ongoing communication with young adults, document communication in the electronic health record, and increase engagement with home blood pressure monitoring. The results of this study will inform a multi-center young adult randomized controlled trial of MyHEART.

## Introduction

In the U.S., over 10 million 18–39 year-olds (1 in 5 men; 1 in 6 women) have hypertension [1,2], increasing their risk of heart failure, stroke, and chronic kidney disease [3,4]. Hypertension control reduces morbidity and mortality [5,6]. Yet only 35% of young adults with hypertension in the U.S. have achieved blood pressure control (< 140/90 mmHg), in contrast to 56% of ≥ 40 year-olds [4].

Traditional hypertension self-management programs targeted towards adults ≥ 50 years old primarily focus on medication titration [7–9]. In contrast, among young adults, a trial of lifestyle modifications is the preferred initial hypertension treatment step for mild hypertension [10]. Unfortunately, current healthcare delivery for hypertension does not routinely provide hypertension self-management counseling (home blood pressure monitoring and lifestyle modifications) and follow-up for young adults [11,12]. Critical provider and patient barriers include limited time to manage multiple co-morbidities and clinic visit non-adherence (young adult clinic no-shows; not scheduling follow-up visits) [13]. Therefore, feasible out-of-clinic self-management support is needed to help overcome these barriers.

To address the unmet need for hypertension care in young adults, we developed MyHEART (My Hypertension Education and Reaching Target), a multi-component intervention founded on the Self-Determination Theory (SDT) [14] designed to achieve hypertension self-management among young adults with uncontrolled hypertension. The intervention was co-designed with young adults with hypertension and primary care providers. To design this intervention, we conducted focus groups of 38 young adults (18–39 year-olds) with hypertension and 9 one-on-one interviews of primary care providers in academic, urban, and rural communities. Through these processes, stakeholders voiced preferences and proposed specific solutions for increasing hypertension self-management education and hypertension control among young adults.

MyHEART incorporates four main components, recommended by the Institute of Medicine [15] and the American Heart Association [16], and implemented by a health coach: 1) telephone-based self-management counseling, 2) home blood pressure monitoring, 3) young adult-focused hypertension education, and 4) electronic health record documentation of coach-participant telephone contacts. MyHEART uses telephone as the primary mode of communication between patients and coaches, because young adults in our focus groups indicated a preference for this mode of delivery over text or in-person visits.

The aim of this 3-month study was to evaluate the feasibility of the MyHEART program in a large multi-specialty academic health system to: 1) effectively recruit young adults with uncontrolled hypertension, 2) maintain ongoing communication between the coach and each young adult throughout the study, 3) increase young adults’ engagement in hypertension self-management (out-of-clinic blood pressure monitoring), 4) effectively document communication in the electronic health record to maintain communication between the patient and his/her healthcare team, and 5) assess patient acceptability.

## Methods

### Participants

This was a nonrandomized feasibility study with no control group. This study was approved as a quality improvement study by the University of Wisconsin Health Sciences Institutional Review Board (IRB) and did not require written informed consent; it was felt that requiring written consent would limit recruitment. However, all potentially eligible participants contacted by the research team were read an IRB-approved project summary to standardize the delivery of patient information. Patients verbally agreed to participate in an audio recorded phone call. Neither patients nor healthcare providers received payment/reimbursement for participation and they were notified of this prior to giving verbal authorization.

Eligible patients were identified via the healthcare system’s electronic health record which had been used in previous studies of this population [10,17]. Inclusion criteria included: 1) 18–39 years old at the start of the study, 2) a minimum of two hypertension ICD-9 coded office visits with any provider (MD, DO, PA, NP) on different dates in the last 24 months, with at least one code in the past 18 months, 3) receiving regular primary care at the multi-specialty group practice per the Wisconsin Collaborative for Healthcare Quality (WCHQ) definition [18], and 4) uncontrolled hypertension (≥ 140/90 mmHg) based on the last ambulatory blood pressure reading. Per WCHQ, patients are defined as receiving regular primary care by a primary care practice if they had two or more billable office encounters in an outpatient, non-urgent primary care setting, or one primary care encounter and one office encounter in an urgent care setting (regardless of diagnosis code), within 3 years, with at least one visit occurring in the prior 2 years [19]. The last ambulatory blood pressure reading had to be within 90 days prior to study start. If multiple blood pressures were recorded on the same day of service, the average of the last two blood pressures on that date was used. Blood pressure readings from inpatient, emergency room, and urgent care visits and self-reported blood pressure readings were excluded. Young adults with uncontrolled hypertension who were prescribed antihypertensive medication were also included since hypertension self-management is part of the treatment program even with medication. To address a frequent limitation in previous studies, patients with diabetes mellitus and chronic kidney disease (stages 1–3) were also included.

**Figure 1:**
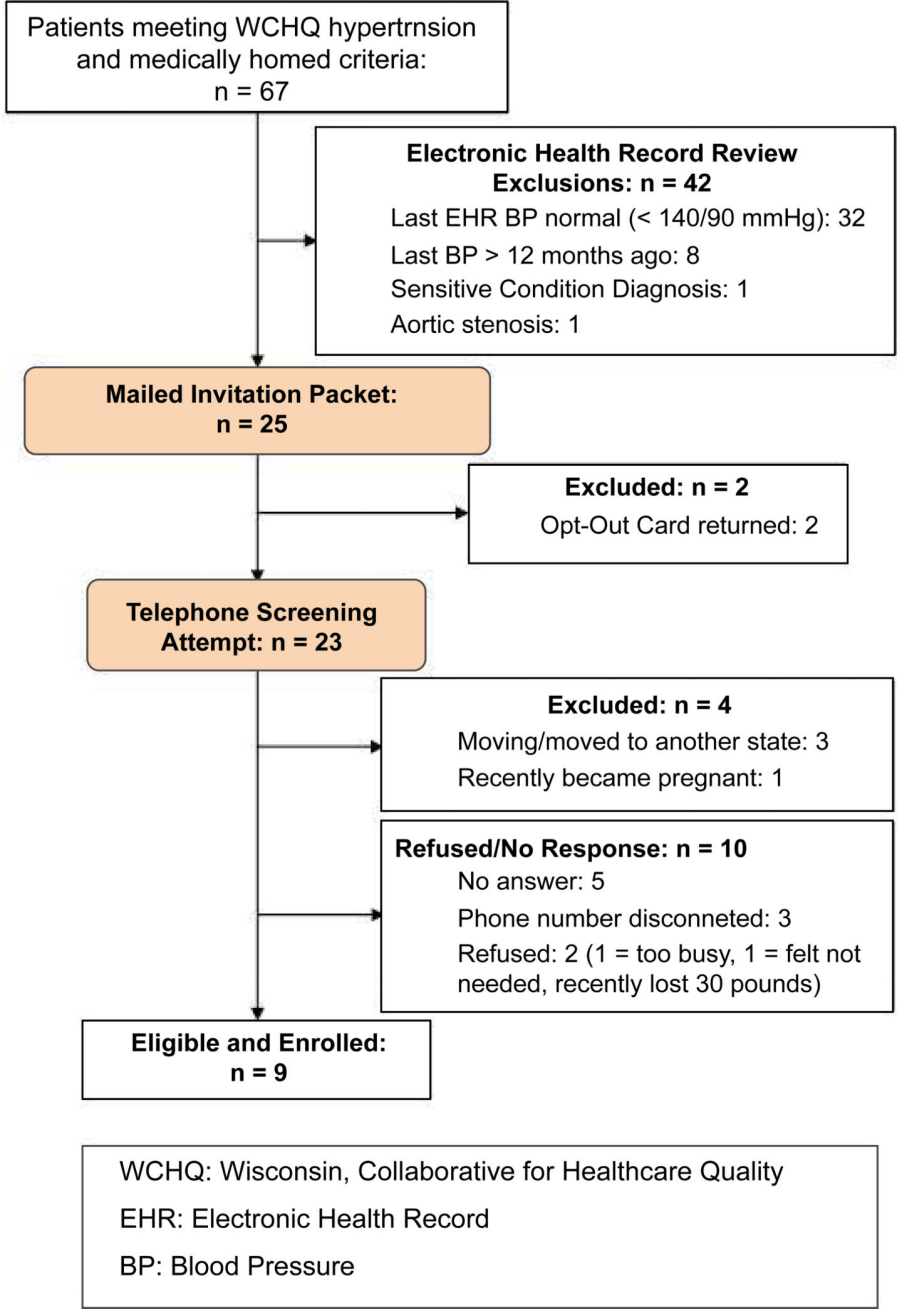
CONSORT - MyHEART feasibility. CONSORT (Consolidated Standards of Reporting Trials) flow diagram of the progress through the phases of patient identification, exclusion, and enrollment.

Exclusion criteria (Table 1) were first assessed by electronic health record indicator variables and also manual electronic health record abstraction. The remaining potentially eligible patients were mailed an introductory MyHEART packet. This packet was pre-approved by young adults in the Community Advisors on Research Design and Strategies (University of Wisconsin Network for Research Support - CARDS^®^) program and primary care leaders of the healthcare system. The packet included: a flyer summarizing the MyHEART program, a pre-paid opt-out postcard, an overview sheet explaining high blood pressure, a handout with instructions on home blood pressure monitoring, a lifestyle modification goal sheet (that would be completed during the program), and a magnet of the MyHEART logo. If an opt-out response was not received by mail or email after 2 weeks, the research coordinator contacted patients to perform a telephone screen of remaining exclusion criteria. Phone numbers were acquired from the electronic health record and were > 98% accurate. Patients who met at least one exclusion criteria were ineligible (Figure 1). Patients who did not answer or return the coordinator’s call after three attempts were also excluded.

### Intervention

During the MyHEART feasibility study, all participants continued to receive usual hypertension care from their primary care provider. The MyHEART intervention involved a health coach calling all eligible and enrolled young adults to perform hypertension self-management telephone counseling. Calls continued every 2 weeks for a total of 6 calls. The interpersonal interaction between the coach and participants in MyHEART was based on the self-determination theory (SDT), which promotes principles consistent with motivational interviewing [20]. The coach’s goal over the 3-month period was to help young adults establish self-management skills.

For this feasibility study, the health coach was a clinical employee within the healthcare system with baseline knowledge of the electronic health record system. Prior to implementing MyHEART, the health coach received 8 hours of training (2 hours, once a week for 4 weeks) led by faculty with experience in behavioral theories and coaching from the University of Wisconsin School of Nursing (Diane Lauver, co-author). This training focused on self-determination theory concepts that overlap with concepts relevant to motivational interviewing [21]. Training included interactive lectures and videos, role playing, and assessment of the coach’s fidelity of protocol delivery with an *a priori* skills checklist, followed by problem solving and debriefing [22,23]. The research team created a MyHEART Health Coach Guide for fidelity of delivery by the coach [22]. The guide included suggested open-ended questions to ask about the target behaviors. The coach promoted autonomy by individualizing the order and depth of educational content based on the behavioral goals chosen by the participant and focusing on patient-identified motives for behavior change [24,25]. In our guide, we specified what a coach should do if participants reported potentially serious symptoms (e.g., chest pain, headache, vision changes, shortness of breath), significantly elevated out-of-clinic blood pressures (systolic blood pressure ≥ 180 mmHg or diastolic blood pressure ≥ 110 mmHg), psychiatric concerns, and/or substance abuse. The coach was to contact the primary care provider or the principal investigator (Heather Johnson, author) immediately. The guide included instructions on when the coach should contact emergency services (i.e., 911), but we had no emergencies.

There were nine self-management modules for MyHEART (Table 2) that were designed based upon our young adult focus groups, previous interventions [25–27], and hypertension guidelines [28]. During the first call, all young adult participants started with the home blood pressure monitoring and hypertension knowledge modules. Home blood pressure feedback was also provided during all follow-up phone calls, which included review of the patient’s blood pressures and discussion of any barriers to home blood pressure monitoring. The order of the remaining modules was guided by the young adult participant’s choice (autonomy) based on their individual goals. All relevant modules were covered during the intervention as applicable (e.g., tobacco cessation only among tobacco users). At the end of the phone calls, the health coach asked participants if they were willing to receive handouts from the MyHEART curriculum to reinforce topics discussed. If they agreed, they had an option of receiving handouts by email or postal mail. At the time of this study, our healthcare system patient portal did not have a means to provide handouts electronically. Some study handouts were identified from national organizations (e.g., the American Heart Association, Centers for Disease Control, National Institutes of Health); others were created by the MyHEART team to include specific information requested by our young adult focus group participants, on topics such as dealing with stress and school-work time management. The MyHEART handouts were formatted with a Flesch-Kincaid readability of ≤ 6^th^ grade [29].

**Figure 2:**
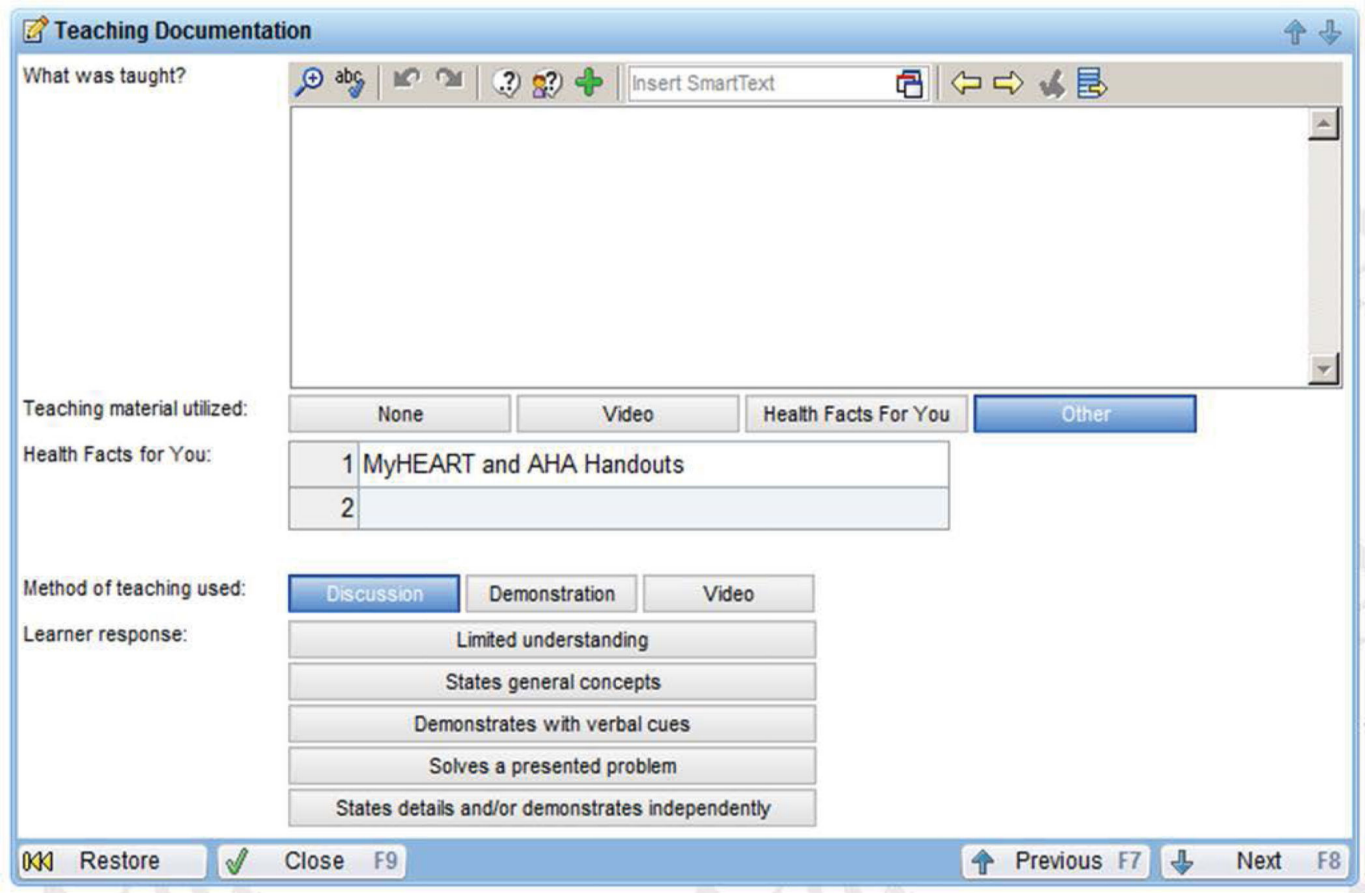
Health coach electronic health record template. Screen capture of the electronic health record lifestyle counseling documentation template. The MyHEART health coach completed a separate entry for each young adult-coach telephone call. The documentation was accessible by the patient’s primary care provider and healthcare team.

All telephone encounters between the health coach and participants were documented by the coach in an electronic health record template (Figure 2; Epic Health Link Electronic Health Record System). This allowed the young adult’s primary care provider and patient care team to review the home blood pressures and topics discussed. All of the coach’s calls were audio recorded with the participant’s recorded verbal permission. Health coach fidelity of the intervention delivery by the coach was evaluated using digital audio-recordings [30] of the contacts and review of their electronic health record documentation. Our fidelity evaluation included assessment of adherence to MyHEART’s intervention protocol [31]. Overall, 10% of each type of call (i.e., baseline, follow-up, final) was randomly selected from all recorded calls. Fidelity data are in-progress and will be reported in a separate manuscript.

### Data collection and analysis

Baseline participant demographic data were abstracted from the electronic health record at the time of study eligibility. Coded responses on the abstraction form were analyzed using descriptive statistics and Microsoft Excel. Insights into young adults’ experiences of the MyHEART program emerged from the comments of the acceptability survey completed at 3-months and direct quotes are provided in the Results [32,33].

## Results

Study enrollment occurred from January 2015-May 2015 and follow-up was for 3 months. As shown in the CONSORT diagram (Figure 1; also see additional file), we screened a total of 67 patients; 42 (62%) were excluded after review of their electronic health record due to achievement of hypertension control (< 140/90 mmHg; n = 11), last blood pressure reading documented more than 90 days since study start (n = 26), a sensitive condition diagnosis (e.g., HIV diagnosis; n = 3), and/or a contraindicated medical condition (e.g., aortic valve stenosis; n = 2). Twenty-five invitation packets were mailed and, of this group, 2 patients (8%) returned opt-out postcards. Telephone screening and enrollment (Jamie LaMantia, co-author) was attempted for the remaining 23 potentially eligible patients: 3 (13%) reported plans to move to another state (exclusion criteria), and 1 (4%) recently became pregnant (exclusion criteria). Ten patients did not respond, could not be reached, or refused: 5 (22%) did not answer the phone, 3 (13%) had a disconnected phone number, 2 (9%) refused and cited reasons of “too busy” (n = 1) and “not needed” (n = 1); this latter patient reported successfully already losing 30 pounds. This resulted in a study uptake of 9 enrolled/19 eligible (47%).

According to table 3, among enrolled patients, the mean (SD) age was 35.8 (2.6) years old, 78% male, 55% White, and 18% Black. The mean (SD) systolic blood pressure at baseline (defined as the last ambulatory blood pressure within 90 days prior to the study) was 141.5 (13) mmHg and the diastolic was 93 (3.8) mmHg. Clinic blood pressures were not assessed at the end of the study since it was not an outcome of this study. The majority of patients (75%) had Internal Medicine primary care providers and 50% of participants lived ≥ 10 miles from their primary clinic. Of the 9 enrolled patients, 8 (89%) maintained communication with the health coach beyond the first call; 1 patient was not able to be reached after the first call.

The median time of the first call was 17.5 minutes (range: 14–20) and each follow-up call duration was a median 14 (range: 9–16) minutes. At the initial call, 7 of the 9 participants (78%) reported receiving the mailed MyHEART introductory packet. Only 1 participant (11%) reported ever checking their blood pressure outside of clinic prior to program enrollment. After the feasibility study, 4 of the 9 participants (44%) reported checking their blood pressure outside of a clinic at least once/week, with home being the usual location.

The health coach was able to document all (100%) telephone encounters in the electronic health record template (Figure 2). Outside of home blood pressure monitoring, the most common topic discussed between the health coach and participants were low sodium/DASH diets and stress management (each discussed in 89% of calls). We received a 44% response rate (n = 4) to our patient satisfaction questionnaire among the 9 enrolled participants. All of the responses were positive and representative responses are provided in table 4. No known harms or unintended effects were reported or identified by the study team. The study was not prematurely ended.

## Discussion

MyHEART is a theoretically-based intervention [34,35] designed to address the low rates of hypertension control among young adults in the U.S. [4]. This multi-component intervention was deemed feasible at this multi-specialty group practice. The identification of potentially eligible participants via the electronic health record was successfully implemented using definitions from our prior studies [10,17]. Since we required recent clinic blood pressures (≤ 90 days) we learned the need to increase the frequency of indicator data queries from the electronic health record to have more up-to-date eligibility data, and we are able to adapt the protocols accordingly.

Study uptake was 47% (9/19) among eligible patients, which is a high enrollment rate [27], and reflects MyHEART’s ability to effectively recruit a challenging, mobile young adult population. However, we had a low representation of the youngest adult age group (18–29 year-olds). One possible reason is that for this feasibility study we were limited to ½ day of the health coach’s time (usually the same day of the week). This would disproportionately impact the youngest age group because of the higher proportion of students and limited availability. In addition, there were fewer eligible within this age group. For the larger randomized controlled trial, additional recruitment steps will include varying the time of day and days of the week for the health coach’s schedule, partnering with primary care providers to review weekly panels, and posting announcements within primary care clinics [36]. Unfortunately, we did not power the study for clinical (blood pressure) outcomes, but the results of this feasibility study will strengthen the design and sample size estimation of our larger, randomized controlled trial.

One significant strength of the MyHEART program is that almost 80% of our population was male. The predominant gender of our study population reflected high prevalence rates of hypertension among young adult males in the U.S. [4]. In addition, we retained our participants; we maintained communication with 89% of the participants after initial enrollment. This could be explained by our preliminary research with young adults to inform the design of the MyHEART program. Our retention rate may be explained by our deliberate focus on meeting interpersonal needs with coaching; we did not “tell them what to do”. However, selection bias during screening/enrollment may also contribute to our higher retention rate which will be addressed in the study design of our larger clinical trial.

Furthermore, because 50% of the participants lived ≥ 10 miles from their primary care clinic, MyHEART can potentially help with transportation barriers associated with hypertension follow-up visits. Although we did not provide home blood pressure monitors for this feasibility study, we had an increase in home blood pressure monitoring from 11% to 44% after health coach phone calls. We were also able to effectively document the coach-patient telephone communications in the electronic health record using standardized templates. For the randomized controlled trial, we will plan to electronically extract data from the electronic health record templates. We received 4 of 9 (44%) patient satisfaction questionnaires. The electronic health record template was designed solely for medical communications and health topics; we were unable to capture additional patient acceptability data from the electronic health record. However, we have edited our template to allow entry of non-medical patient comments and subjective data.

In a larger study, considerations for scalability will include the cost of mailing follow-up patient education handouts; additionally, mailing handouts does not ensure patients will receive the information, given this population’s greater likelihood of transition between residences. Our focus will be to increase the portability of our patient education via a MyHEART website, email, and increased use of the electronic health record patient portal, with future study designs including video-conferencing (e.g. Skype) and additional mobile health technology. Although we did not assess provider’s acceptability of this program, we received unsolicited feedback about physician’s satisfaction of being able to review and reinforce hypertension topics discussed by the health coach. Most importantly, we had outstanding patient acceptability comments across gender and ethnicity about the MyHEART program which supports the generalizability of this study. We look forward to ongoing patient and stakeholder engagement as we transition to a randomized controlled trial.

## Conclusions

This study demonstrated that MyHEART was feasible for delivery by the healthcare system and acceptable to young adults with uncontrolled hypertension. MyHEART can successfully recruit young adults, a hard-to-reach population. Health coaches can effectively maintain ongoing communication with these young adults, document this communication in the electronic health record, and increase young adult’s engagement in home blood pressure monitoring. The findings from this preliminary study also highlight the need to increase blood pressure follow-up for young adults with uncontrolled hypertension and promote effective communication with their healthcare team. The results of this study will inform a multi-center young adult randomized controlled trial of MyHEART that addresses these intervention goals.

## Figures and Tables

**Table 1:** MyHEART feasibility exclusion criteria

**Data Source: Electronic Health Record - Hypertension Registry and** **Manual Abstraction**
Chronic Kidney Disease (Stage 4 or 5 or Dialysis)
Congestive Heart Failure, Any Etiology
Activated Healthcare Power of Attorney
Skilled nursing facility or correctional facility residence
Currently enrolled in case management or chronic disease management support services
Sensitive condition diagnosis (e.g., HIV)
Prescribed warfarin, novel oral anticoagulant, or insulin
**Data Source: Telephone Screen Self-report)**
Sickle cell anemia or cystic fibrosis
Stroke, myocardial infarction, coronary artery revascularization
Syncope within past 12 months
Prior or planned organ transplant
Chemotherapy or radiation therapy within 6 months
Severely impaired hearing or speech
Current participation in another research study
Pregnant/planning to become pregnant in the next 12 months
Planning to leave the area in the next 3 months
Any health condition that will limit physical activity or diet
Illegal drug use (other than marijuana) in the past 30 days
Unable to read or communicate in English

**Table 2:** MyHEART feasibility young adult education modules.

Module	Topic Overview
Home Blood Pressure Monitoring	How to measure blood pressure at home (or outside of clinic)
Hypertension Knowledge	Define blood pressure, hypertension, and goal blood pressure
Low Sodium	Reading labels, effects of sodium on blood pressure
Dietary Approaches to Stop Hypertension (DASH) Eating Plan	DASH components, meal planning
Weight Loss/Maintenance	Relationship of weight with hypertension, dietary and activity options to lose weight, time management
Smoking Cessation	Negative effects of tobacco on heart health, tobacco cessation information for the Wisconsin Quit Line
Moderate Alcohol Consumptions	Negative effects of high alcohol consumption on heart health; Primary care provider notification for addiction services consultation if needed
Blood Pressure Medicine	Why blood pressure medications may be part of the treatment plan
Social Support	Local resources to reduce no-shows and for community activity options
Stress Management	Stress with chronic disease and life stressors

**Table 3:** MyHEART feasibility baseline demographics (n = 9).

**Age, *m (SD)***	35.8 (2.6)
**Male, *n (%)***	7 (78%)
**Race (self-report), *n (%)***	
White	5 (56%)
Black	3 (33%)
Other	1 (11%)
**Baseline SBP, mmHg, *m (SD)***	141.5 (13)
**Baseline DBP, mmHg, *m (SD)***	93 (3.8)
**Primary Care Clinic, *n (%)***	
Internal Medicine	7 (77%)
Family Medicine	2 (23%)
**Distance from Primary Clinic, *n (%)***	
< 10 miles	5 (55%)
10–30 miles	2 (22.5%)
> 30 miles	2 (22.5%)

**Table 4:** Sample patient acceptability comments from the MyHEART feasibility study.

Question	Free Text Survey Responses
The things I liked best about the MyHEART program were:	**38 -year-old male:** “I have lower blood pressures and fewer headaches.”
What was it about the MyHEART program that was most helpful to your goals?	**37-year-old male:** “Phone calls make me accountable. It is now in my head to make good decisions.”
What was it about the MyHEART program that motivated you?	**36-year-old male:** “I don’t want to die young and I want to be here for my family.”

## Abbreviations

MyHEART: My Hypertension Education and Reaching Target
SDT: Self-Determination Theory
IRB: Institutional Review Board
WCHQ: Wisconsin Collaborative for Healthcare Quality
CARDS^®^: Community Advisors on Research Design and Strategy (a program through the University of Wisconsin Network for Research Support)
DASH: Dietary Approaches to Stop Hypertension

## References

[1] http://www.cdc.gov/bloodpressure/facts.htm

[2] Nguyen QC, Tabor JW, Entzel PP, Lau Y, Suchindran C (2011). Discordance in national estimates of hypertension among young adults. Epidemiology.

[3] Mitchell AB, Cole JW, McArdle PF, Cheng YC, Ryan KA (2015). Obesity increases risk of ischemic stroke in young adults. Stroke.

[4] Mozaffarian D, Benjamin EJ, Go AS, Arnett DK, Blaha MJ (2016). Heart Disease and Stroke Statistics-2016 Update: A Report From the American Heart Association. Circulation.

[5] Chobanian AV, Bakris GL, Black HR, Cushman WC, Green LA (2003). The Seventh Report of the Joint National Committee on Prevention, Detection, Evaluation, and Treatment of High Blood Pressure: the JNC 7 report. JAMA.

[6] Ford ES, Ajani UA, Croft JB, Critchley JA, Labarthe DR (2007). Explaining the decrease in U.S. deaths from coronary disease, 1980–2000. N Engl J Med.

[7] Bosworth HB, Powers BJ, Olsen MK, McCant F, Grubber J (2011). Home blood pressure management and improved blood pressure control: results from a randomized controlled trial. Arch Intern Med.

[8] Jackson GL, Oddone EZ, Olsen MK, Powers BJ, Grubber JM (2012). Racial differences in the effect of a telephone-delivered hypertension disease management program. J Gen Intern Med.

[9] Margolius D, Bodenheimer T, Bennett H, Wong J, Ngo V (2012). Health coaching to improve hypertension treatment in a low-income, minority population. Ann Fam Med.

[10] Johnson HM, Thorpe CT, Bartels CM, Schumacher JR, Palta M (2014). Antihypertensive medication initiation among young adults with regular primary care use. J Gen Intern Med.

[11] http://www.ahrq.gov/research/findings/final-reports/ptmgmt/ptmgmt.pdf

[12] Michie S, Abraham C, Whittington C, McAteer J, Gupta S (2009). Effective techniques in healthy eating and physical activity interventions: a meta-regression. Health Psychol.

[13] Walsh JM, Sundaram V, McDonald K, Owens DK, Goldstein MK (2008). Implementing effective hypertension quality improvement strategies: barriers and potential solutions. J Clin Hypertens (Greenwich).

[14] Deci EL, Ryan RM (1985). Intrinsic Motivation and Self-Determination in Human Behavior.

[15] Institute of Medicine (US) Committee on Public Health Priorities to Reduce and Control Hypertension (2010). A Population-Based Policy and Systems Change Approach to Prevent and Control Hypertension.

[16] Go AS, Bauman MA, Coleman King SM, Fonarow GC, Lawrence W (2014). An effective approach to high blood pressure control: a science advisory from the American Heart Association, the American College of Cardiology, and the Centers for Disease Control and Prevention. J Am Coll Cardiol.

[17] Johnson HM, Thorpe CT, Bartels CM, Schumacher JR, Palta M (2014). Undiagnosed hypertension among young adults with regular primary care use. J Hypertens.

[18] Sheehy AM, Flood GE, Tuan WJ, Liou JI, Coursin DB (2010). Analysis of guidelines for screening diabetes mellitus in an ambulatory population. Mayo Clin Proc.

[19] Thorpe CT, Flood GE, Kraft SA, Everett CM, Smith MA (2011). Effect of patient selection method on provider group performance estimates. Med Care.

[20] Markland D, Ryan RM, Tobin VJ, Rollnick S (2005). Motivational interviewing and self-determination theory. J Soc Clin Psychol.

[21] Miller WR, Rose GS (2009). Toward a theory of motivational interviewing. Am Psychol.

[22] Bellg AJ, Borrelli B, Resnick B, Hecht J, Minicucci DS (2004). Enhancing treatment fidelity in health behavior change studies: best practices and recommendations from the NIH Behavior Change Consortium. Health Psychol.

[23] Madson MB, Loignon AC, Lane C (2009). Training in motivational interviewing: a systematic review. J Subst Abuse Treat.

[24] Glasgow RE, Funnell MM, Bonomi AE, Davis C, Beckham V (2002). Self-management aspects of the improving chronic illness care breakthrough series: implementation with diabetes and heart failure teams. Ann Behav Med.

[25] Gutnick D, Reims K, Davis C, Gainforth H, Jay M (2014). Brief action planning to facilitate behavior change and support patient self-management. J Clin Outcomes Manag.

[26] Bosworth HB, Olsen MK, Neary A, Orr M, Grubber J (2008). Take Control of Your Blood Pressure (TCYB) study: a multifactorial tailored behavioral and educational intervention for achieving blood pressure control. Patient Educ Couns.

[27] Poobalan AS, Pitchforth E, Imamura M, Tucker JS, Philip K (2009). Characteristics of effective interventions in improving young people’s sexual health: a review of reviews. Sex Educ.

[28] Weber MA, Schiffrin EL, White WB, Mann S, Lindholm LH (2014). Clinical practice guidelines for the management of hypertension in the community: a statement by the American Society of Hypertension and the International Society of Hypertension. J Clin Hypertens (Greenwich).

[29] http://www.dtic.mil/cgi-bin/GetTRDoc?AD=ADA006655

[30] Antal H, Hossain MJ, Hassink S, Henry S, Fuzzell L (2015). Audio-video recording of health care encounters for pediatric chronic conditions: observational reactivity and its correlates. J Pediatr Psychol.

[31] Johnson-Kozlow M, Hovell MF, Rovniak LS, Sirikulvadhana L, Wahlgren DR (2008). Fidelity issues in secondhand smoking interventions for children. Nicotine Tob Res.

[32] Forster DA, Savage TL, McLachlan HL, Gold L, Farrell T (2014). Individualised, flexible postnatal care: a feasibility study for a randomised controlled trial. BMC Health Serv Res.

[33] Pope C, Ziebland S, Mays N, Pope C, Mays N (2006). Analysing Qualitative Data. Qualitative Research in Health Care.

[34] Michie S, Abraham C (2004). Interventions to change health behaviours: evidence-based or evidence inspired?. Psychol Health.

[35] Michie S, Prestwich A (2010). Are interventions theory-based? Development of a theory coding scheme. Health Psychol.

[36] Rocha-Goldberg Mdel P, Corsino L, Batch B, Voils CI, Thorpe CT (2010). Hypertension Improvement Project (HIP) Latino: results of a pilot study of lifestyle intervention for lowering blood pressure in Latino adults. Ethn Health.

[37] Ogrinc G, Davies L, Goodman D, Batalden P, Davidoff F (2015). SQUIRE 2.0 Standards for QUality Improvement Reporting Excellence: revised publication guidelines from a detailed consensus process. BMJ Qual Saf.

